# Manual of Physiology

**Published:** 1849-10

**Authors:** 


### REVIEWS.

---

ART. VI.—*Manual of Physiology*. By W. SENHOUSE KIRKES, M. D. Assisted by JAMES PAGET, Lecturer on General Anatomy and Physiology, at St. Bartholomew's Hospital, with 118 illustrations on wood. Philadelphia: Lea & Blanchard. 12mo. pp. 548. 1849.

We remember once to have heard an eminent medical professor discoursing to a popular audience on the circulation of the blood, and at the close of his lecture his hearers must have been sadly puzzled to determine by what power it is that the vital fluid is kept in motion. It could not be the heart, for the learned professor assured them that that organ was sometimes found "ossified," and yet the blood continued to move. It could not be the arteries, for no contraction was observed to take place in them. What, then, could it be? This was a problem which was left unsolved by the veteran lecturer. Our authors do not appear to have found it so embarrassing, nor will the reader be at a loss to account for the circulation, after a careful perusal of their chapter on the subject. In the muscular substance of the heart, they rationally find the principal force for moving the blood; and, as assistant forces, they mention the elastic walls of the arteries, the pressure of the muscles among which some of the veins run, and the movements of the walls of the chest in respiration. If the heart is not designed mainly for the propulsion of the blood, what, we fancy the reader is ready to inquire, is its office in the animal economy? And yet it is not probable that it maintains the motion of the blood in its entire course, for the capillaries are capable of accelerating or arresting it.—"That the movement of the blood through the arterial trunks and the capillary tubes is, in man, and in other warm-blooded animals, chiefly dependent upon the action of the heart, there can be no doubt whatever," says Carpenter. But, as remarks this writer, "certain residual phenomena indicate that this is not the whole truth, even in man." A circulation is maintained in plants and in the lower animals, independently of a heart. In cold-blooded animals, the complete excision of the heart does not put a stop to the movement of the blood in the capillaries; and cases are on record, in which the functions have gone on healthily in the human fetus during the whole of embryonic life, the heart being absent. "A single case of this kind," observes Dr. Carpenter," furnishes all the proof that can be needed, of the existence, even in the highest animals, of a capillary power; which, though usually subordinate to the heart's action, is sufficiently strong to maintain the circulation by itself, when the power of the central organ is diminished."

What is this power of the capillaries? It is not mechanical, for these vessels, when examined microscopically, present no obvious movement, and the stream of blood "rendered continuous by the elasticity of the arteries, passes through them as through unelastic tubes." It is chemical, and has been well illustrated by Prof. Draper, in his work on the Food of Plants. If, he says, two liquids communicate with each other through a capillary tube, for the walls of which they both have an affinity, and if this affinity is stronger in the one liquid than in the other, a movement will ensue; the liquid which has the greatest affinity being absorbed most energetically into the tube, and driving the other before it. The same result occurs when the fluid is drawn, not into a single tube, but into a network of tubes, permeating a solid structure —for if this porous structure be previously saturated with the fluid, for which it has the less degree of attraction, this will be driven out, and replaced by that for which it has the greater affinity, when the latter is permitted to enter it. Now if, in its passage through the porous solid, the liquid undergo such a change, that its affinity be diminished, it is obvious that, according to the principle just explained, it must be driven out by a fresh supply of the original liquid; and that thus a continual movement in the same direction would be produced.

This, he continues, is probably what seems to take place in the organized tissue, permeated by nutritious fluid. This fluid and the solid matter through which it is distributed, have a certain affinity, which is exercised in the nutritive changes to which the fluid becomes subservient during the course of its circulation. And Dr. Carpenter applies this principle, thus, to the circulation in the capillaries:

The arterial blood,—containing oxygen with which it is ready to part, and being prepared to receive in exchange the carbonic acid which the tissues set free,—must obviously have a greater affinity for the tissues, than venous blood; in which both these changes have already been effected. Consequently, upon mere physical principles, the arterial blood which enters the systemic capillaries on one side, must drive before it, and expel on the other side of the net-work, the blood which has become venous whilst traversing it. But if the blood which enters the capillaries have no such affinity, no such motor power can be developed.

On the other hand, in the capillaries of the lungs the opposite affinities prevail. The venous blood and the air in the pulmonary cells have a mutual attraction, which is satisfied by the exchange of oxygen and carbonic acid that takes place through the walls of the capillaries; and when the blood has become arterialized, it no longer has any attraction for the air. Upon the very same principle, therefore, the venous blood will drive the arterial before it, in the pulmonary capillaries, whilst respiration is properly going on: but if the supply of oxygen be interrupted, so that the blood is no longer aerated, no change in the affinities takes place whilst it traverses the capillary network; the blood continuing venous still retains its need of a change, and its attraction for the walls of the capillaries; and so its egress into the pulmonary veins is thus resisted, rather than aided, by the force generated in the lungs.

The change in the condition of the blood, in regard to the relative proportions of its oxygen and carbonic acid, is the only one to which the pulmonary circulation is subservient; but in the systemic circulation, the changes are of a much more complex nature;—every distinct organ attracting to itself the peculiar substances which it requires as the materials of its own nutrition; and the nature of the affinities thus generated being consequently different in each case. But the same law holds good in all instances. Thus the blood conveyed to the liver by the portal vein, contains the materials at the expense of which the bile-secreting cells are developed; consequently the tissue of the liver, which is principally made up of these cells, possesses a certain degree of affinity or attraction for blood containing these materials; and this is diminished, so soon as they have been drawn from it into the cells around. Consequently the blood of the portal vein will drive before it, into the hepatic vein, the blood which has traversed the capillaries of the portal system, and which has given up, in doing so, the elements of bile to the solid tissues of the liver. The same principle holds good in every other case.

This "assistant force," we think it is clear, must be added to the motive powers of the circulation enumerated by our authors. In the lower animals, it may be, and probably is, the "principal" force; and in man and the higher orders, "whilst the injection of blood *into* the capillary vessels of every part of the system is due to the action of the heart, its rate of passage *through* those vessels is greatly modified by the degree of activity in the processes, to which it should normally be subservient in them ; the current being rendered more rapid by an increase in their activity, and being stagnated by their depression or total cessation." Thus it appears that " the capillaries possess a *distributive* power over the blood, regulating the local circulation, independently of the central organ, in obedience to the necessities of each part"—a power beautifully explained by the principle of Prof. Draper just alluded to.

In regard to the rythmic movements of the heart, Dr. Kirkes justly holds that the cause must reside in the organ itself, and independent of the stimulus of the blood. These movements persist after the removal of the heart from the body. From the experiments of Remak, Volkmann and Dr. Robert Lee, he thinks it probable that the cause of this action is " connected with the existence of numerous minute ganglia of the sympathetic nervous system, which, with connecting nerve-fibres, are distributed through the substance of the heart." He continues—

These ganglia appear to act as so many centres or organs for the production of motor impulse ; while the connecting nerve-fibres unite them into one system, and enable them to act in concert and direct their impulses so as to excite in regular series the successive contractions of the several muscles of the heart. The mode in which ganglia thus act as centres and co-ordinators of nervous power will be described in the chapter on the Nervous System ; and it will appear probable that the chief peculiarity of the heart, in this regard, is due to the number of its ganglia, and the apparently equal power which they all exercise ; so that there is no one part of the heart whose action, more than another's, determines the actions of the rest. Thus, if the heart of a reptile be bisected, the rythmic successive actions of auricle and ventricle will go on in both halves ; we therefore cannot say that the action of the right side determines or regulates that of the left, or vice versa ; and we must suppose that when they act together in the perfect heart, it is because they are both, as it were, set to the same time. Neither can we say that the auricles determine the action of the ventricles ; for, if they are separated, they will both contract and dilate in regular, though not necessarily similar, succession. A fact pointed out by Mr. Malden shows how the several portions of each cavity are similarly adjusted to act alike, yet independently of each other. If a point of the surface of the ventricle of a turtle's or frog's heart be irritated, it will immediately contract, and very quickly afterwards all the rest of the ventricle will contract; but, in the close of this general contraction, the part that was irritated and contracted first, is slightly distended or pouched out, showing that it was adjusted to contract in and for only a certain time, and that therefore as it began to contract first, so it first began to dilate.

The influence of other organs upon the heart, our authors explain, not by "sympathy," but upon the principle of nervous connection—chiefly the sympathetic and pneumogastric nerves ; and this influence, they properly maintain, is proved in a much more striking manner by the phenomena of disease, than by any experimental or physiological observations.

The influence of a shock in arresting or modifying the action of the heart,—its very slow action after compression of the brain, or injury to the cervical portion of the spinal cord,—its irregularities and palpitations in dyspepsia and hysteria,—are better evidences for the connection of the heart with the other organs, through the nervous system, than any results obtained by experiments. The best of such results are recorded by E. H. Weber (xv. Art. *Muskelbewegung*), who found that the electro-magnetic stimulus applied in the frog to the *bulbus arteriosus*, around which the principal fibres of the sympathetic nerves supplying the heart are placed, accelerated and strengthened the heart's action ; but, applied to the pulsating part of the vena cava inferior, where are the principal filaments it derives from the pneumogastric nerves, retarded the action. He is disposed, therefore, to think that, in general, stimuli conveyed through the sympathetic nerve would accelerate, and through the pneumogastric would retard, the heart's action. The latter conclusion is corroborated by the fact, also stated by him, that the heart's action is retarded by stimulus applied to any part between the corpora quadrigemina and posterior part of the fourth ventricle of the brain, or to both trunks of the pneumogastric nerves at once, or by division of both pneumogastric nerves in the necks of Mammalia.

One great advantage of the elasticity of the arteries is that it produces an equable movement of the blood—the force with which they are dilated every time the ventricles contract, being "received by them in store to be all given out again in the next succeeding period of dilatation of the ventricles;" so that in the capillaries and veins the intermittent accelerations produced in the arterial current, by the action of the heart, cease to be observable. Other purposes served by the elasticity of the arteries, are the increased capacity which they thus acquire for receiving more than the average quantity of blood under particular circumstances, and their ability to adapt themselves to the different movements of the several parts of the body.

The *muscularity* of arteries has been disputed by some physiologists of late, which leads our authors to go at length into the evidence for it, which we quote:

When a small artery in the living subject is exposed to the air or cold, it gradually but manifestly contracts.—Hunter (i. vol. iii. p. 157) observed that the posterior tibial artery of a dog when laid bare became in a short time so much contracted as almost to prevent the transmission of blood; and the observation has been often and variously confirmed. Simple elasticity could not effect this; for after death, when the vital muscular power has ceased, and the mechanical elastic one alone operates, the contracted artery dilates again.

When an artery is cut across its divided ends contract, and the orifices may be completely closed. The rapidity and completeness of this contraction are different in different animals; they are generally greater in young than in old animals; and less, apparently, in man than in animals. The contraction is generally increased by the application of cold, or of any simple stimulating substances, or by mechanically irritating the cut ends of the artery, as by pricking or twisting them. Such irritation would not be followed by these effects if the arteries had no other power of contracting than that depending upon elasticity.

The contractile property of arteries continues many hours after death, and thus affords an opportunity of distinguishing it from elasticity. When a portion of an artery, for example, the splenic artery, of a recently killed animal, is exposed, it gradually contracts, and its canal may be thus completely closed: in this contracted state it remains for a time, varying from a few hours to two days—then it dilates again, and permanently retains the same size. If, while contracted, the artery be forcibly distended, its contractility is destroyed, and it holds a middle or natural size.

This persistence of the contractile property after death was well shown in an observation of Hunter, which may be mentioned as proving also the greater degree of contractility possessed by the smaller than the larger arteries. Having injected the uterus of a cow, which had been removed from the animal upwards of twenty-four hours, he found, after the lapse of another day, that the larger vessels had become much more turgid than when he injected them, and that the smaller arteries had contracted so as to force the injection back into the larger ones.

The results of an experiment which Hunter made with the vessels of an umbilical cord, proves still more strikingly, the long continuance of the contractile power of arteries after death. In a woman delivered on a Thursday afternoon, the umbilical cord was separated from the fetus, having been first tied in two places, and then cut between, so that the blood contained in the cord and placenta was confined in them. On the following morning, Hunter tied a string round the cord, about an inch below the other ligature, that the blood might still be confined in the placenta and remaining cord. Having cut off this piece, and allowed all the blood to escape from its vessels, he attentively observed to what size the ends of the cut arteries were brought by the elasticity of their coats, and then laid aside the piece of cord to see the influence of the contractile power of its vessels. On Saturday morning, the day after, the mouths of the arteries were completely closed up. He repeated the experiment the same day with another portion of the same cord, and on the following morning found the results to be precisely similar. On the Sunday, he performed the experiment the third time, but the artery seemed then to have lost its contractility, for, on the Monday morning, the mouths of the cut arteries were found open. In each of these experiments there was but little alteration perceived in the orifices of the veins (i. vol. iii. p. 158).

The influence of cold in increasing the contraction of a divided artery has been referred to: it has been shown, also, by Schwann, in an experiment on the mesentery of a living toad. Having extended the mesentery under the microscope, he placed upon it a few drops of water, the temperature of which was some degrees lower than that of the atmosphere. The contraction of the vessels soon commenced, and gradually increased until, at the expiration of ten or fifteen minutes, the diameter of the canal of an artery, which at first was 0.0724 of an English line, was reduced to 0.0276. The arteries then dilated again, and at the expiration of half an hour had acquired nearly their original size. By renewing the application of the water, the contraction was reproduced: in this way the experiment could be performed several times on the same artery. The veins did not contract. It is thus proved that cold will excite contraction in the walls of very small as well as of comparatively large arteries: it could not produce such contraction in a merely elastic substance; but it is a stimulus to the organic muscular fibres in many other parts, as well as in the arterial coats; as, e. g. in the walls of the bronchi, and the dartos.

Lastly, satisfactory evidence of the muscularity of the arterial coats is furnished by the experiments of Ed. and E. H. Weber (lxxx. 1847, p. 232), in which they applied the stimulus of electro-magnetism to small arteries. One principal circumstance which induced Müller to deny the muscularity of arteries, was the seeming impossibility of producing contractions in arteries by galvanic and electric stimuli, which excite all true muscular tissues to manifest contraction. An explanation of the failure may be found in the circumstance that, in nearly all the experiments, the arteries examined were of large size, such as the aorta and the carotids, in which there is little or no muscular tissue. The experiments of the Webers were performed on the small mesenteric arteries of frogs ; and the most striking and satisfactory results were obtained, when the diameter of the vessels examined did not exceed from one-seventh to one-seventeenth of a Paris line. When a vessel of this size was exposed to the electric current, its diameter, in from five to ten seconds, became one-third less, and the area of its section about one-half. On continuing the stimulus, the narrowing gradually increased, until the calibre of the tube became from three to six times smaller than it was at first, so that only a single row of blood corpuscles could pass along it at once ; and eventually the vessel was closed and the current of blood arrested.

Thus, with the exception of the largest trunks, the arteries appear to offer every necessary evidence of muscularity. One of their coats has the structure and chemical composition of organic muscle ; and, like such muscle they contract on exposure, on division and mechanical irritation, after death with a kind of *rigor mortis*, on the application of cold, and under the stimulus of electricity: in all these contractions they are reduced to a size less than that which their mere elasticity would give them, and from all they are commonly, after a time, again by their elasticity restored to a larger size.

The purposes served by the muscular coat of the arteries are two-fold ;—it assists in propeling the onward current of blood ; and first limits and then arrests its flow when an artery is divided.

The most probable office of the muscular coat is that of regulating the quantity of blood to be received by each part, and of adjusting it to the requirements of each according to various circumstances ; but, chiefly and most naturally, according to the activity with which the functions of each part are at different times performed. The amount of function discharged by each organ of the body varies at different times, and the variations often quickly succeed each other, so that, like the brain, for example, during sleep and waking, within the same hour, a part may be now very active and then inactive. In all its active exercise of function, such a part requires a larger supply of blood than is sufficient for it during the times when it is comparatively inactive. It is evident that the heart cannot regulate the supply to each part at different periods, neither could it be regulated by any general and uniform contraction of the arteries; but it may be regulated by the power which the arteries of each part have, in their muscular coat, of contracting so as to diminish, and of dilating to increase, the supply of blood according to the need. And thus, while the ventricles of the heart determine the total quantity of blood to be sent onwards at each contraction, and the force of its propulsion, and while the large and merely elastic arteries distribute it and equalize its stream, the smaller arteries with muscular coats add to these two purposes, that of regulating and determining the proportion of the whole quantity of blood which shall be distributed to each part.

On the nature and function of the capillaries, our authors remark—

Microscopic observations and minute injections have shown that the capillary vessels are merely the fine tubes which form the medium of transition from arteries to veins, and that no other kind of vessel arises from them; that the minute arteries have no other mode of termination than the communication with the veins by means of the capillaries; in a word, that there are no exhalant or other vessels terminating by open extremities. The hypothesis of the existence of such vessels is alike unnecessary to the explanation of secretion, growth, nutrition, and all the other functions of a part. All that these functions require of the vessels is, that, for each tissue, the blood should be brought so near to the active elements of the tissue, that some of its fluid part may be absorbed or imbibed by them. And the distance through which such imbibition is effected is not always small; in the brain, for example, those portions of its substance which lie in the middle of the wide meshes of the capillary network must be nourished by imbibing fluid through the spaces between them and the nearest vessels; so also in bone, where the vessels are yet wider apart. But the instances in which such imbibition is effected through the greatest distance are in what are called non-vascular tissues, which receive no bloodvessels into their own substance, and are nourished by the fluid absorbed from the vessels of the adjacent vascular parts; as the cornea from the vessels of the conjunctiva, the lens from those of the posterior layer of its capsule, articular cartilage from the vessels of the subjacent bone. The difference of nutrition in vascu lar and non-vascular tissues is only one of degree; in all parts alike the elementary structures are outside the vessels, and obtain new materials from the blood by imbibition; but the imbibition has to be accomplished through a greater distance in the less than in the more vascular parts.

The rate of the circulation has occupied the attention of physiologists, at different times, and seems to have been accurately determined by some recent experiments. The velocity of the blood's movement, as Müller has justly observed, is not to be judged of by the rapidity with which it flows from a vessel when divded. The rate, in this case, is the result of the entire pressure to which the whole mass of blood is subjected in the vascular system, and which meets with no resistance at the point of the incision; whilst in the closed vessels no portion of blood can be moved forwards but by the propulsion of the whole mass, and by overcoming the resistance growing out of the friction in the smaller vessels. Experiments to ascertain the rapidity with which poisons introduced into the blood are transmitted from one part of the vascu lar system to another, have afforded data for a very satisfactory estimation of the velocity of the blood in its motion through the body.

From eighteen such experiments on horses, Hering deduced that the time required for the passage of a solution of ferrocyanide of potassium, mixed with the blood, from one jugular vein (through the right side of the heart, the pulmonary circulation, the left cavities of the heart, and the general circulation) to the jugular vein of the opposite side, varies from twenty to thirty seconds. The same substance was transmitted from the jugular vein to the great saphena in twenty seconds; from the jugular vein to the masseteric artery in between fifteen and thirty seconds, to the facial artery in one experiment in between ten and fifteen seconds, in another experiment in between twenty and twenty-five seconds: in its transit from the jugular vein to the metatarsal artery it occupied between twenty and thirty seconds, and in one instance more than forty seconds. The result was nearly the same whatever was the rate of the heart's action.

Poiseuille's observations (xxxi. 1843) accord completely with the above; and show, moreover, that when the ferrocyanide is injected into the blood with other substance, such as acetate of ammonia, or nitrate of potash, (solutions of which, as other experiments have shown, pass quickly through capillary tubes) the passage from one jugular vein to the other is effected in from eighteen to twenty-four seconds; while if, instead of these, alcohol is added, the passage is not completed until from forty to forty-five seconds after injection. Still greater rapidity of transit has been observed by Mr. J. Blake (xciv. Oct., 1841), who found that nitrate of baryta injected into the jugular vein of a horse could be detected in blood drawn from the carotid artery of the opposite side in from fifteen to twenty seconds after the injection. In sixteen seconds a solution of nitrate of potash, injected into the jugular vein of a horse, caused complete arrest of the heart's action, by entering and diffusing itself through the coronary arteries. In a dog, the poisonous effects of strychnia on the nervous system were manifested in twelve seconds after injection into the jugular vein; in a fowl, in six and a half seconds, and in a rabbit in four and a half seconds.

In all these experiments, it is assumed that the substance injected moves with the blood, and at the same rate as it, and does not move from one part of the organs of circulation to another by diffusing itself through the blood or tissues more quickly than the blood moves. The assumption is so probable that it may be considered nearly certain that the times above mentioned, as occupied in the passage of the injected substances, are those in which the portion of blood into which each was injected was carried from one part to another of the vascular system. It would, therefore, appear that a portion of blood can traverse the entire course of the circulation, in the horse, in half a minute; of course it would require longer to traverse the vessels of the most distant part of the extremities than to go through those of the neck; but taking an average length of vessels to be traversed, and assuming, as we may, that the movement of blood in the human subject is not slower than in the horse, it may be concluded that one minute, which is the estimate usually adopted of the average time in which the blood completes its entire circuit in man, is rather above than below the actual rate.

The next topic discussed by our authors is, the "*peculiarities of the circulation in different parts*," the most remarkable of which are presented by the lungs, the liver, the brain and the erectile organs. Whether the brain contains at one time more blood than at another, is a question, the reader needs not to be informed, which has divided the ablest physiologists and pathologists of the day. The functions of the brain require that it should receive a large supply of blood. It is also necessary that the force with which the blood is sent to the brain should be subject to less variation from external circumstances than it is in other parts. Uniformity in the supply of blood is a third condition indispensabble to the integrity of its functions, and required by the character of the matter of which it is composed.

The experiments of Dr. Kellie appeared to establish the position that the quantity of blood in the brain must be at all times the same; and that the quantity received, in a given time by the arteries, must be always, and at the same time, exactly equal to that removed by the veins. According to his observation, in animals bled to death, while all the other organs of the body were nearly emptied of blood, the vessels of the brain contained almost their ordinary quantity; but he found that if, previous to bleeding an animal, he made a hole in its cranium, thus exposing the brain equally with the other organs to the influence of atmospheric pressure, its vessels, like those of other parts of the body, were emptied as the fatal hemorrhage proceeded.

But Dr. Burrows, as we showed, in a former number, having repeated these experiments, and performed others in addition, has obtained different results. He found that in animals bled to death the brain became pale and anemic without the aid of an aperture in the cranium. He also found evidences of congestion of the cerebral vessels, in rabbits, killed by strangling or drowning; and from all his experiments he concluded,

That although the total volume of the contents of the cranium is probably nearly always the same, yet the quantity of blood in it is liable to variation, its increase or diminution being accompanied by a simultaneous diminution or increase in the quantity of the cerebro-spinal fluid, which, by readily admitting of being removed from one part of the brain and spinal cord to another, and of being rapidly absorbed, and as readily effused, would serve as a kind of supplemental fluid to the other contents of the cranium, to keep it uniformly filled in case of variations in their quantity (see also Ecker, cxxix). But such variations occur only in abnormal conditions; in ordinary states, and in health, it is probable that the arrangements of the vessels, to which we have referred, insure to the brain a supply of blood which is both uniform and guarded from the accidental disturbances to which the supply for all other organs is liable even in circumstances consistent with health.

Respiration is the next subject considered, and is taken up here rather than among the excretory functions, because of its intimate relation to the circulation. The blood, as it circulates through the various parts of the body, not only loses a part of its nutritive properties, but becomes charged with impurities, resultiug from the disintegration of the tissues. One of the most abundant of these is carbonic acid, the removal of which, and the introduction of fresh supplies of oxygen, are the great ends of respiration, of which a very full and satisfactory account is given by our authors.

The quantity of air changed in the lungs in each act of ordinary breathing varies according to circumstances and in different individuals, but is correctly enough stated by Coathupe at from twenty to twenty-five cubic inches. In old people, according to Bourgery, breathing is deeper, and the quantity of air changed in each respiration greater. Hutchison calls the air habitually changed in breathing, *breathing air ;* that which remains after ordinary expiration, he calls *reserve air;* and he names that *supplemental air*, which a man can draw into the lungs in the deepest inspiration. But after the most violent expiratory efforts a certain quantity of air always remains in the lungs, which our athors propose to call *residual air.* The respiratory capacity of the chest, indicated by the quantity of air which a person can expel from the lungs by a forcible expiration after the deepest breathing, is termed by Hutchison the *vital capacity*, and expresses the power which the individual possesses in the emergencies of active exercise, violence, and disease. This capacity varies with the age, stature, and weight of individuals. For a man five feet seven inches high, at sixty degrees Fahrenheit, according to this author, it is two hundred and twenty five cubic inches. For every inch above this standard, we learn from the same writer, the capacity is increased, on an average, by eight cubic inches; and for every inch below, it is diminished by the same amount. It is singular that this relation of capacity to height should be "quite independent of the absolute capacity of the cavity of the chest," so that a person of small absolute capacity of chest may have a large capacity of respiration, and the reverse. The capacity of respiration depends upon "the mobility of the walls of the chest"; but why this mobility should increase in a definite ratio with the height of the body, as remarked by our authors, is yet unexplained; and is the more difficult of solution, as the height of the body is determined more by the length of the legs than by that of the trunk.

The lungs have been shown, by modern anatomists, to possess "organic-muscular circular fibres," in the larger branches of the bronchial tubes, from which they derive "some power of spontaneous contraction." In ordinary breathing these fibres do not appear to be put in requisition, nor indeed is it known under what circumstances they are brought into action. Their office, our authors believe, "is to regulate and adapt, in some measure, the quantity of air admitted to the lungs, and to each part of them, according to the supply of blood." This muscular action, morbidly excited, they conjecture, "is the chief cause of the phenomena of spasmodic asthma."

Changes take place in the air inspired essential, as is well known, to the continuance of life, and it is so ordered that the movements of respiration go on uninterruptedly when we are asleep and unconscious, as well as during our waking hours—that this great function is forgetful of repose. The first act of the new born child is to inhale the vital air; the last effort of the expiring man is to command its receding wave. Not only is the process involuntary, but it is aided by physical laws which operate as well upon inanimate mattar as in the living systems of animals. The air itself assists, by certain qualities impressed upon it, in the respiratory movements, having in view changes in its condition.

5*

According to the law observed in the diffusion of gases, the carbonic acid evolved in the air-cells will, independently of any respiratory movement, tend to leave the lungs, by diffusing itself into the external air where it exists in less proportion; and, according to the same law, the oxygen of the atmospheric air will tend of itself towards the air-cells in which its proportion is less than in the air in the bronchial tubes or external to the body.—But for this tendency of the oxygen and carbonic acid to mix uniformly, within and without the lungs, the *reserve* and *residual* air would, probably, be very injuriously charged with carbonic acid; for the respiratory movements alone are not enough to empty the air-cells, and perhaps expel only the air which lies in the larger bronchial tubes. Probably also the change is assisted by the different temperature of the air within and without the lungs; and by the action of the ciliæ on the mucous membrane of the bronchial tubes, the continual vibrations of which may serve to prevent the adhesion of the air to the moist surface of the membrane.

Regarding the quantity of carbon excreted from the lungs in respiration, writers on the subject are not agreed. Andral and Gavarret state it at nine ounces per day; Coathupe makes it scarcely five ounces; while Liebig computed the quantity that escapes from the lungs and skin at 13.9 ounces. Some of these discrepancies, as our authors remark, are no doubt due to the variations to which the exhalation of carbonic acid is liable in different circumstances.

The quantity of oxygen consumed in respiration is greater than is shown in the carbonic acid exhaled from the lungs, in the proportion of 1583 to 1345 cubic inches, or nearly 635 grains, in the hour. This waste goes, probably, to form the carbonic acid given off by the skin, and to oxidise the phosphorus and sulphur, which are excreted as phosphates and sulphates in the urine. Valentin and Brunner have attempted to show that the exchanged quantities of oxygen and carbonic acid are always in proportion to their *diffusion volumes;* but there are weighty objections to so stringent a law. If the exchange of gases were according to this law, we should have none of those variations which the varying condition of the general economy seems to render necessary; the proportions would remain inflexibly fixed. But this is not the case, and, in the nature of living things, ought not. Besides, as objected by our authors, "the conditions of the gases engaged in respiration are not those in which the law of diffusion would exactly hold." They are not free and under equal pressure; and, no doubt, this explains the deviations from the law, "while it is really in operation."

It has been a question whether carbonic acid existed in the blood, but all doubts respecting it have been removed by the experiments of Magnus, who has shown that it, as well as oxygen, is mingled with the fluid, both in the arteries and veins. This experimenter has also shown, that the relative proportions of these gases (now no longer in the gaseous state) differ in the two kinds of blood. In that of the arteries, the quantity of oxygen is twice as great as in venous blood, or ten per cent. of the volume of the former, and only about five per cent. of the volume of the latter. On the other hand, the quantity of carbonic acid is less in arterial than in venous blood, "amounting to about twenty volumes per cent. in the former, and twenty-five per cent. in the latter."

Till the existence of the gases in the blood was clearly proved, the theory most favored was, that the oxygen of the atmospheric air permeates the membranous walls of the air-cells, enters the blood, and there at once combines with carbon derived from the disintegrated tissues, to form carbonic acid, which escapes, together with the greater part of the nitrogen previously absorbed from the atmosphere. It could be well objected, even when the existence of gases in the blood was doubtful, that if this theory were true, the lungs ought to be much warmer than other parts of the body, through the quantity of heat given out in the quick union of the carbon with the oxygen of the atmosphere; and that such was not found to be the case; the temperature of the blood in the left side of the heart being never more than one or two degrees higher than that in the right.

It has not yet been decided whether part, or the whole of the oxygen imbibed is at once united chemically with any of the constituents of the blood; but our authors incline to the opinion that the greater portion is held in solution "by the fluid part of the blood," and that the emission of carbonic acid and the imbibition of oxygen are effected upon the general plan of secretion and absorption of fluids by other organs, not by the diffusion-process just adverted to. The reaction between these gases, they hold to be the great source of *animal heat*, concerning which they remark–

Many things observed in the economy and habits of animals are explicable by this theory, and are, therefore, evidence for its truth. Thus, as a general rule, in the various classes of animals, as well as in individual examples of each class, the quantity of heat generated in the body is in direct proportion to the activity of the respiratory process. The highest animal temperature, for example, is found in birds, in whom the function of respiration is most actively performed. In Mammalia, the process of respiration is less active, and the average temperature of the body less, than in birds. In reptiles, both the respiration and the heat are at a much lower standard; whilst in animals below them, in which the function of respiration is at the lowest point, a power of producing heat is, in ordinary circumstances, hardly discernible. Among these lower animals, however, the observations of Mr. Newport (xliii. 1837) supply confirmatory evidence. He shows that the larva, in which the respiratory organs are smaller in comparison with the size of the body, has a lower temperature than the perfect insect. Volant insects have the highest temperature, and they have always the largest respiratory organs and breathe the greatest quantity of air : while among terrestrial insects, those also produce the most heat which have the largest respiratory organs and breathe the most air. During sleep, hybernation, and other states of inaction, respiration is slower or suspended, and the temperature is proportionally diminished ; while on the other hand, when the insect is most active, and respiring most voluminously, its amount of temperature is at its maximum, and corresponds with the quantity of respiration. Neither the rapidity of the circulation, nor the size of the nervous system, according to Mr. Newport, presents such a constant relation to the evolution of heat.

Similar evidence in favor of this theory of animal heat is furnished by the fact that heat is sometimes evolved by plants, in a quantity which appears to be in direct proportion to the amount of oxygen they at the same time absorb and convert into carbonic acid. For example, their evolution of heat is most evident during flowering and the germination of seeds, the times at which the largest amount of carbonic acid is exhaled.

The quantity and quality of food consumed by man and animals in the different climates and seasons, also appears to be adapted to the production of various amounts of heat by the combination of carbon and hydrogen with oxygen. In northern regions, for example, and in the colder seasons of more southern climes, the quantity of food consumed is (speaking very generally) greater than is consumed by the same men or animals in opposite conditions of climate and season. And the food which appears naturally adapted to the inhabitants of the coldest climates, such as the several fatty and oily substances, abounds in carbon and hydrogen, and is fitted to combine with the large quantities of oxygen which, breathing cold dense air, they absorb from their lungs.

We propose to continue our notice of this interesting volume, in a future number.

